# Impacts of ainuovirine-based and efavirenz-based antiretroviral therapies on the lipid profile of HIV/AIDS patients in southern China: a real-world study

**DOI:** 10.3389/fmed.2023.1277059

**Published:** 2024-01-08

**Authors:** Quan Zhang, Zhong Chen, Yating Wang, Yongquan Peng, Si Tan, Ying Li, Guiying Cao, Antonia Bignotti, Shangjie Wu, Min Wang

**Affiliations:** ^1^Department of Pulmonary and Critical Care Medicine, The Second Xiangya Hospital, Central South University, Changsha, China; ^2^Hunan Centre for Evidence-based Medicine, Changsha, China; ^3^Institute of HIV/AIDS The First Hospital of Changsha, Changsha, China; ^4^Graduate Collaborative Training Base of the First Hospital of Changsha, Hengyang Medical School, University of South China, Hengyang, China; ^5^Department of Pathology and Laboratory Medicine, The University of Kansas Medical Center, Kansas City, KS, United States

**Keywords:** human immunodeficiency virus, ainuovirine, efavirenz, lipid profile, efficacy

## Abstract

**Background:**

The newly approved third-generation oral anti-HIV-1 drug, ainuovirine (ANV), was used in combination with nucleoside reverse transcriptase inhibitors (NRTIs) in our study, and its effects on the lipid profile of antiretroviral-experienced HIV/AIDS patients are unclear.

**Objectives:**

This study aimed to examine the effects of antiretroviral agents on the lipid profile in patients with HIV/AIDS.

**Methods:**

We conducted a real-world prospective study involving treatment-naive and treatment-experienced adult participants living with HIV-1 infection provided with ANV- or efavirenz (EFV)-based regimens. The primary endpoint was the proportion of participants with an HIV-1 RNA level of <50 copies/mL at week 24 of treatment. Secondary endpoints included the change from baseline in CD4+ T-cell count and lipid profile.

**Results:**

A total of 60 treatment-naive and 47 treatment-experienced participants received an ANV-based regimen, while 88 treatment-naive and 47 treatment-experienced participants receiving an EFV-based regimen were, respectively, matched as controls. At week 24 following treatment, the proportion of participants with an HIV-1 RNA level of <50 copies/mL and the mean changes of CD4+ T-cell counts from baseline were significantly higher in naive-ANV group than those in naive-EFV group (*p* < 0.01). Compared with the EFV group, both naive and experienced ANV groups exhibited a favorable lipid profile, including constant changes in total cholesterol and triglycerides, a significant decrease in LDL-cholesterol (*p* < 0.0001), and a dramatic increase in HDL-cholesterol (*p* < 0.001).

**Conclusion:**

The efficacy of ANV was non-inferior to EFV when combined with two NRTIs. Patients receiving ANV-based regimens had a decreased prevalence of dyslipidemia.

## 1 Introduction

Human immunodeficiency virus (HIV)/acquired immunodeficiency syndrome (AIDS) has been a long-standing global health challenge, with an estimated 38.4 million people living with HIV (PWH) and 650 thousand AIDS-related deaths reported by the Joint United Nations Program on HIV/AIDS in 2021. Over the past three decades, significant progress has been made in the research and development of novel antiviral drugs, with a particular focus on antiretroviral therapy (ART). According to the World Health Organization guidelines for HIV treatment, initial ART regimens should include a nucleoside reverse transcriptase inhibitor (NRTI) backbone, combined with either dolutegravir (DTG) or efavirenz (EFV) as alternatives (1). Based on the most recent Chinese guidelines, recommended first-line regimens comprise two NRTIs combined with a third class of agent (2). With the fast virologic responses and persistent suppression in this population, the advances in the treatment of HIV/AIDS have led to a significant improvement in both life expectancy and quality of life for the affected individuals. Nonetheless, a broad prevalence of dyslipidemia in PWH undergoing combination ART (c-ART), which is considered to be the most important risk factor of atherosclerotic cardiovascular disease (ASCVD), has always puzzled patients and medical workers. Such a group of patients may experience an increased risk of non-HIV/AIDS causes of disease, which include vascular complications, posing new clinical challenges. EFV is the only non-NRTI (NNRTI) that has an adverse effect in raising low-density lipoprotein cholesterol (LDL-C) when compared to other NNRTIs such as nevirapine and rilpivirine (3). Hence, it highlights the importance of developing a new generation of NNRTIs that are as effective as EFV but have fewer side effects. As a result, a novel NNRTI, ainuovirine (ANV), has been developed to meet this need and received approval for treating HIV-1 infection in China in 2021. A phase III trial has reported on the 96-week efficacy and safety of ANV with lamivudine/tenofovir disoproxil fumarate in treatment-naive HIV-1-positive adults (4).

However, the data published to date remain incomplete due to little data on lipid profile disorders associated with ANV used in antiretroviral-experienced HIV/AIDS patients. As a clinical trial center for ANV, our institute prospectively conducted a comprehensive and longitudinal assessment of virologic and immunologic responses as well as lipid profile changes in both treatment-naive and treatment-experienced HIV/AIDS patients receiving ANV-based regimens in comparison with two groups of matched HIV/AIDS patients receiving EFV-based regimens. The aim of this study was to assess the effectiveness and practicality of transitioning from EFV to ANV with lamivudine (3TC) and tenofovir disoproxil fumarate (TDF) in two subgroups of HIV-1-positive adult patients within a real-world clinical setting.

## 2 Patients and methods

### 2.1 Study design and patients

The Institutional Review Board of the Chinese Center for Disease Control and Prevention approved the study (No. X220314682). This study was conducted among treatment-naive and treatment-experienced HIV-infected patients who attended and were followed up at the Institute of HIV/AIDS, First Hospital of Changsha, China. Patients receiving an ANV-based regimen who attended the clinic between March 2022 and October 2022 were assessed for eligibility and enrolled when they met the following inclusion criteria: (1) patients older than 17 years with a confirmed diagnosis of HIV/AIDS, including those who had never taken antiretroviral (ARV) drugs (treatment-naive) or who were currently taking antiretroviral drugs (treatment-experienced); (2) patients who were prescribed to take an ANV-based regimen by the infectious disease doctors and thus were divided into the naive-ANV group and the experienced-ANV (exp-ANV) group, respectively; (3) patients who were followed up at 12-week intervals on average; and (4) HIV-infected patients who constantly and regularly maintained the assigned treatment regimen at least during the study period. This study excluded patients who were pregnant or breastfeeding, and those who had severe hepatic impairment, renal failure, severe immunological diseases, psychiatric illness, or active tuberculosis co-infection. Two matched control groups [naive-EFV group: without treatment before; and experienced-EFV (exp-EFV) group: already on ARV-based regimen] who attended our clinic during 2020–2022 were managed according to the standard of care under the guidelines. As an extension of an optional sub-study of the multi-center clinical trial (ChiCTR1800019041) (4), this real-world study mainly assessed the impact of ANV on lipid profile. Written informed consent was obtained from each patient.

### 2.2 Procedures

Participants in the trial groups received a once-daily oral therapy comprised of either 150 mg ANV + 300 mg 3TC + 300 mg TDF (both naive and experienced ANV groups), while patients in the control groups (both naive and experienced EFV groups) received 600 mg EFV + 300 mg 3TC + 300 mg TDF at night before bedtime.

### 2.3 Measurements

The levels of total cholesterol (TC) and triglycerides (TG) were determined using COP-CE-PAP and GPO-PAP assays, respectively (Hunan Yonghe-Sun Biotechnology Co. Ltd., China). The levels of high-density lipoprotein cholesterol (HDL-C) and LDL-C were both measured using catalase scavenging assays (Ningbo Ruiyuan Biotechnology Co., Ltd., China). The inter- and intra-assay coefficients of variation for all parameters were <3.1% and <3.0%, respectively. Plasma HIV-RNA levels were determined using the branched-chain DNA (b-DNA) technique (Chiron, Inc.: detection limit of 50 copies/ml), and CD4+/CD8+ T-cell counts by means of an elite flow cytometer (Coulter Corporation, Miami, FL). All the other clinical measurements were routinely completed in the Clinical Department of Laboratory Medicine.

### 2.4 Data collection and outcomes

All medical records of the enrolled patients were carefully collected and individually reviewed by two members of our team. We recorded the demographics, underlying diseases, and clinical and laboratory parameters of all patients. The primary efficacy endpoints were the percentage of participants with HIV-RNA levels ≤50 copies/mL at week 24 of treatment, and changes in the CD4+ T-cell count and lipid profile at week 12 and 24 of treatment. Safety outcomes were recorded as the incidence of adverse events (AEs), which occurred during 12 weeks and 24 weeks in both study periods, including symptoms, vital signs, and the change of laboratory values. The analysis especially focused on NNRTI mechanism-based AEs, including the incidence of rash and neuropsychiatric events, the severity of drug-induced hepatitis, and changes in fasting serum lipids profile from baseline to week 24. All clinical outcomes were extracted and determined by investigators who were blinded to the therapeutic interventions.

### 2.5 Statistics

Continuous variables were expressed as means ± standard deviations or medians ± interquartile ranges (IQRs) as appropriate. We implemented propensity score matching (PSM) using the R package “MatchIt” version 4.1.0 with the following settings: 1:2 (for naive-EFV group) and 1:1 (for exp-EFV group) pairing, nearest-neighbor methods, and a caliper of 0.1 to balance the age, sex, body weight index (BMI), and baseline lipid levels (TC and TG) of patients with ANV-based and EFV-based treatments. A paired or an unpaired Student’s *t*-test or a Wilcoxon signed-rank test and a Mann–Whitney U test were used for comparison between the two groups, depending on the distribution of the data. All categorical variables were described as the number or percentage, and Fisher’s exact or chi-squared test was used for statistical analysis. All tests were two-tailed, and a *p*-value of <0.05 was considered to be statistically significant. Statistical analysis was performed using SPSS 26.0 (IBM, Armonk, NY, United States), or the GraphPad Prism 9.4 software (San Diego, CA, United States).

## 3 Results

### 3.1 Patient characteristics

This prospective study recruited a cohort of 69 treatment-naive and 55-experienced PWH who received ANV. After matching, 60 of 69 in the naive-ANV group and 88 of 131 in the naive-EFV group PWH were enrolled in the naive group study. Similarly, after matching,47 of 55 in the exp-ANV group and 47 of 83 in the exp-EFV group PWH were enrolled in the experienced group study, respectively. In total, 92% of the experienced patients were previously taking 600 mg EFV + 300 mg 3TC + 300 mg TDF regimen, the most commonly used first-line ART regimen for adults and adolescents.

There was no significant difference in demographic features between the two groups by age, sex, body mass index (BMI), ethnicity, or comorbidity. As shown in Tables 1, 2, the majority of participants were male (78.3% vs. 81.8% in naive groups; 80.9% vs. 83.0% in experienced groups). The four groups (naive-ANV vs. naive-EFV and exp-ANV vs. exp-EFV) exhibited similar prevalence of diabetes mellitus, hypertension, and hyperlipidemia, as well as percentage of smokers. In terms of transmission route, the ANV-based groups, taking naive-ANV and exp-ANV together, had a relatively higher rate of heterosexual participants (36.4% on ANV vs. 25.9% on EFV, *p* = 0.08) but a significantly lower rate of homosexual participants (17.8% on ANV vs. 53.3% on EFV, *p* < 0.001) than those in the EFV-based groups. Again, regardless of the naive or experienced patients, there were no significant differences between the ANV-based groups and the EFV-based groups concerning the baseline CD4+ T-cell counts, CD4+/CD8+ T-cell count ratios, and plasma HIV-RNA levels (Tables 1, 2).

**Table 1:** Baseline characteristics between matched treatment-naive patients receiving ANV-based and EFV-based treatments.

Characteristics	Naive-ANV patients (*n* = 60)	Naive-EFV patients (*n* = 88)	*p*-value
Sex (male/female)	47/13	72/16	0.60
Age, year	47 (32, 53)	42 (33, 54)	0.76
BMI (kg/m^2^)	21.9 ± 2.5	22.0 ± 1.9	0.63
Smoking, *n* (%)	39 (65.0)	61 (69.3)	0.58
**Ethnicity**			1.00
Han, *n* (%)	60 (100)	86 (97.7)	–
Others, *n* (%)	0 (0)	2 (2.3)	–
**Transmission route**			
Homosexual	9 (15.0)	43 (48.9)	<0.001-
Heterosexual	29 (48.3)	25 (28.4)	0.01-
unknown	22 (36.7)	20 (22.7)	0.07-
**Comorbidity**			
Diabetes mellitus (*n*, %)	5 (8.3)	11 (12.5)	0.43
Hypertension (*n*, %)	13 (14.8)	12 (13.6)	0.20
Hyperlipidemia (*n*, %)	7 (8.0)	14 (15.9)	0.47
**Laboratory baseline**			
CD4+ T-cell count (cells/μL)	212 (58, 410)	249 (181, 354)	0.18
<200, *n* (%)	28 (46.7)	28 (31.8)	–
>200, *n* (%)	32 (53.3)	60 (68.2)	–
CD4+/CD8+ T-cell ratio (1.5–2.5)	0.23 (0.09, 0.36)	0.26 (0.17, 0.35)	0.10
Plasma HIV-1 RNA (copies/mL), *n* (%)			0.64
<100,000	38 (63.3)	59 (67.0)	–
>100,000	22 (36.7)	29 (33.0)	–
TC (2.33–5.69)	4.0 (3.5, 4.5)	4.1 (3.4, 4.8)	0.61
TG (0.25–1.71)	1.4 (1.1, 2.0)	1.4 (1.1, 2.0)	0.44
HDL-C (0.90–1.94)	1.0 (0.9, 1.3)	1.4 (1.0, 1.7)	0.01
LDL-C (0.6–4.14)	2.3 (1.7, 2.7)	3.8 (2.6, 4.5)	0.01

Data are presented as median, interquartile range, or *n* (%). *p* values comparing two groups were obtained from the χ^2^ test, Fisher’s exact test, or the Mann–Whitney U test. *p* < 0.05 means a statistically significant difference between the trial and control groups. BMI, body mass index; total cholesterol, TC; triglyceride, TG; high-density lipoprotein cholesterol, HDL-C; low-density lipoprotein cholesterol, LDL-C.

**Table 2:** Baseline characteristics between matched treatment-experienced patients receiving ANV-based and EFV-based treatments.

Characteristics	Exp-ANV patients (*n* = 47)	Exp-EFV patients (*n* = 47)	*p*-value
Sex (male/female)	38/9	39/8	0.79
Age, year	38 (31, 49)	35 (28, 48)	0.29
BMI (kg/m^2^)	23.0 (20.4, 24.8)	23.2 (22.0,24.0)	0.79
Smoking, *n* (%)	9 (19.1)	17 (36.2)	0.07
Years on ART	3 (1, 6)	2 (2, 2)	0.01
**Ethnicity**			1.00
Han, *n* (%)	46 (97.9)	45 (95.7)	
Others, *n* (%)	1 (2.1)	2 (4.3)	
**Transmission route**			
Homosexual	10 (21.3)	29 (61.7)	<0.001
Heterosexual	10 (21.3)	10 (21.3)	1.00
Unknown	27 (57.4)	8 (17.0)	<0.001
**Comorbidity**			
Diabetes mellitus (*n*, %)	3 (6.4)	3 (6.4)	1.00
Hypertension (*n*, %)	5 (10.6)	5 (10.6)	1.00
Hepatitis (*n*, %)	2 (4.3)	0 (0)	0.21
Hyperlipidemia (*n*, %)	4 (8.5)	8 (17.0)	0.22
**Laboratory baseline**			
CD4+ T-cell count (cells/μL)	500 (267, 694)	489 (363, 579)	0.73
<200, *n* (%)	10 (21.3)	0 (0)	
>200, *n* (%)	37 (76.4)	47 (100)	
CD4+/CD8+ T-cell ratio (1.5–2.5)	0.56 (0.40, 0.73)	0.50 (0.40, 0.60)	0.40
Plasma HIV-1 RNA (copies/mL), *n* (%)			0.24
<10,000	44 (93.6)	47 (100)	
>10,000	3 (6.4)	0 (0)	
TC (2.33–5.69)	4.7 (4.0, 5.2)	4.6 (4.0, 5.2)	0.49
TG (0.25–1.71)	1.7 (1.1, 2.7)	1.5 (1.2, 2.7)	0.60
HDL-C (0.90–1.94)	1.1 (0.9, 1.3)	1.3 (1.1, 1.8)	0.01
LDL-C (0.6–4.14)	2.8 (2.1, 3.6)	1.7 (1.3, 2.7)	<0.001

Data are presented as median, interquartile range, or *n* (%). *p* values comparing two groups were obtained from the χ^2^ test, Fisher’s exact test, or the Mann–Whitney U test. *p* < 0.05 means a statistically significant difference between the trial and control groups. BMI, body mass index; ART, antiretroviral therapy; total cholesterol, TC; triglyceride, TG; high-density lipoprotein cholesterol, HDL-C; low-density lipoprotein cholesterol, LDL-C.

The safety and efficacy of ANV combination therapies with lamivudine/tenofovir disoproxil fumarate at 48 weeks of treatment have been demonstrated for treatment of treatment-naive HIV-1-positive adults (4). Similarly, throughout the 24 weeks of observation in our study, no drug-induced renal injury or incidence of rash was reported except for a slight elevation in alanine transaminase (8.3% on naive-ANV vs. 15.9% on naive-EFV; 12.7% on Exp-ANV vs. 17.0% on Exp-EFV) (*p* > 0.05), neuropsychiatric events of insomnia (5% on naive-ANV vs. 8.0% on naive-EFV, *p* = 0.71; 0% on exp-ANV vs. 23.4% on exp-EFV, *p* < 0.001), and dizziness (6.7% on naive-ANV vs. 8.0% on naive-EFV, *p* = 0.77; 0% on exp-ANV vs. 25.5% on exp-EFV, *p* < 0.001).

### 3.2 HIV-1 RNA level and CD4+ T cell changes after treatment in the naive and experienced HIV/AIDS patients

**Figure 1:**
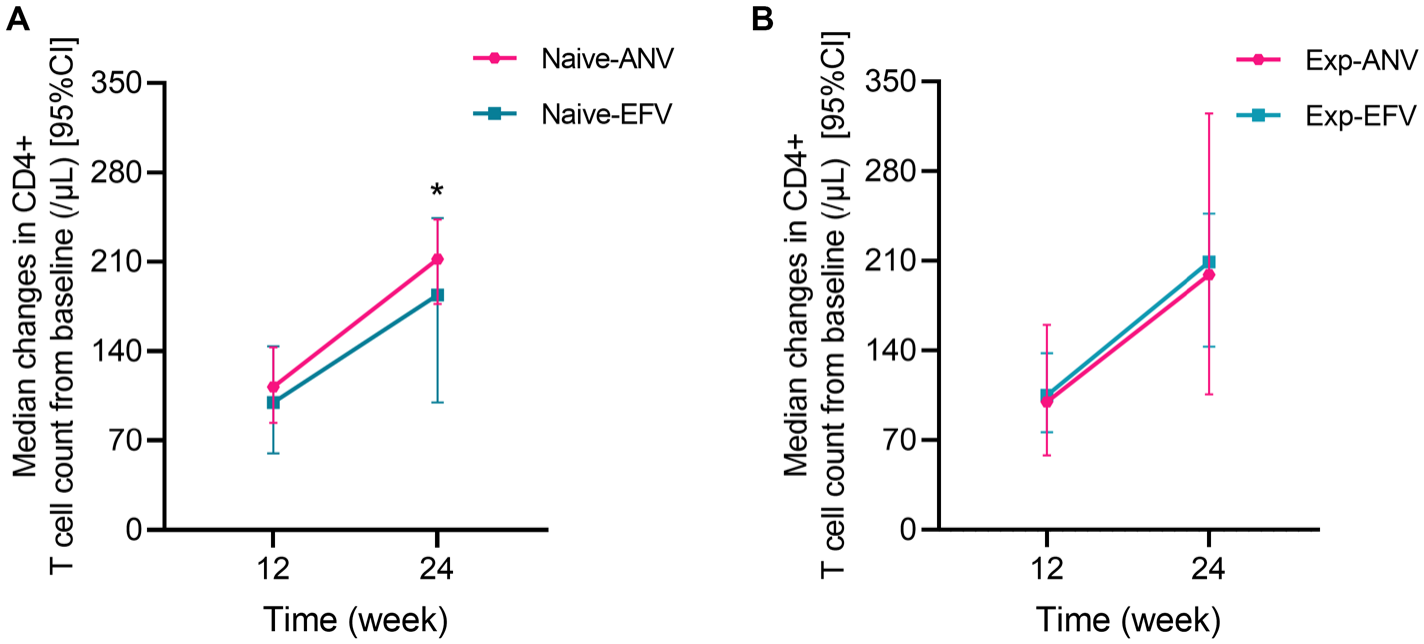
Median changes in CD4+ T cell count from baseline over time among treatment-naive and experienced patients receiving ainuovirine (ANV) or efavirenz (EFV)-based antiretroviral therapy. Median changes in CD4+ T cells means the median of differential values between the baseline level and T12 or T24 (median of =T12w-baseline, T24w-baseline). **(A)** shows the median changes of CD4+ T cells over time in treatment-naive patients. **(B)** shows the median changes of CD4+ T cells over time in treatment-experienced patients.

We assessed the HIV-1 RNA levels at 24 weeks after treatment initiation as the primary endpoint. The results revealed that a significantly higher percentage of participants achieved an HIV-1 RNA level of less than 50 copies/mL in the naive-ANV group. Specifically, 58 of 60 participants (96.7%) in the naive- ANV group and 67 of 88 participants (76.1%) in the naive-EFV group achieved this outcome (*p* = 0.001). Unexpectedly, of those patients with a load of HIV-1 RNA >100,000 copies/mL in the naive-ANV group, 20 of 22 (90.9%) accomplished complete viral suppression, whereas in the naive-EFV group, only 16 of 29 (55.2%) achieved complete viral suppression (*p* = 0.006). Furthermore, significant differences were also observed between the two groups even among patients with low initial viral loads (<100,000 copies/mL). The ANV-based regimen demonstrated superiority in this subgroup, with 100% of participants achieving complete viral suppression, compared with 86.4% (51/59) of participants in the EFV-based groups (*p* = 0.02). The median change of CD4+ T-cell counts from baseline to endpoint was significantly higher in the naive-ANV group (median, 212; IQR, 177–243 cells/μL) than in the naive-EFV group (185; IQR, 100–245 cells/μL) during the observational week of 24 (*p* = 0.04), but not at week 12 (113; IQR, 84–143 cells/μL vs. 100; IQR, 60–144 cells/μL) (*p* = 0.28) (Figure 1A). In addition, among participants with a baseline CD4+ T-cell count of <200 cells/μL, the proportion of participants with CD4+ T-cell counts increasing to above 200 cells/μL at week 24 was significantly higher in the naive-ANV group than that in the naive-EFV group (96.4% in ANV vs. 74.1% in EFV) (p = 0.02), but lower at week 12 (53.6% in ANV vs. 82.1% in EFV) (*p* = 0.02). Again, since all were treatment-experienced patients, only 8 in the exp-ANV group had a load of >400 copies/mL HIV-1 RNA before the treatment, and only one case with the load bounced back to 510 copies/mL at week 24. HIV-1 RNA levels of all patients in the exp-EFV group were kept constantly below 50 copies/mL or not detected. Regarding the changes in the CD4+ T-cell count, these two groups did not show significant superiority or inferiority (Figure 1B). Collectively, these results demonstrate that the ANV-based regimen is at least non-inferior or even superior to EFV-based regimens in both treatment-naive and treatment-experienced PWH.

### 3.3 Lipid level changes after treatment in the naive HIV/AIDS patients

**Figure 2:**
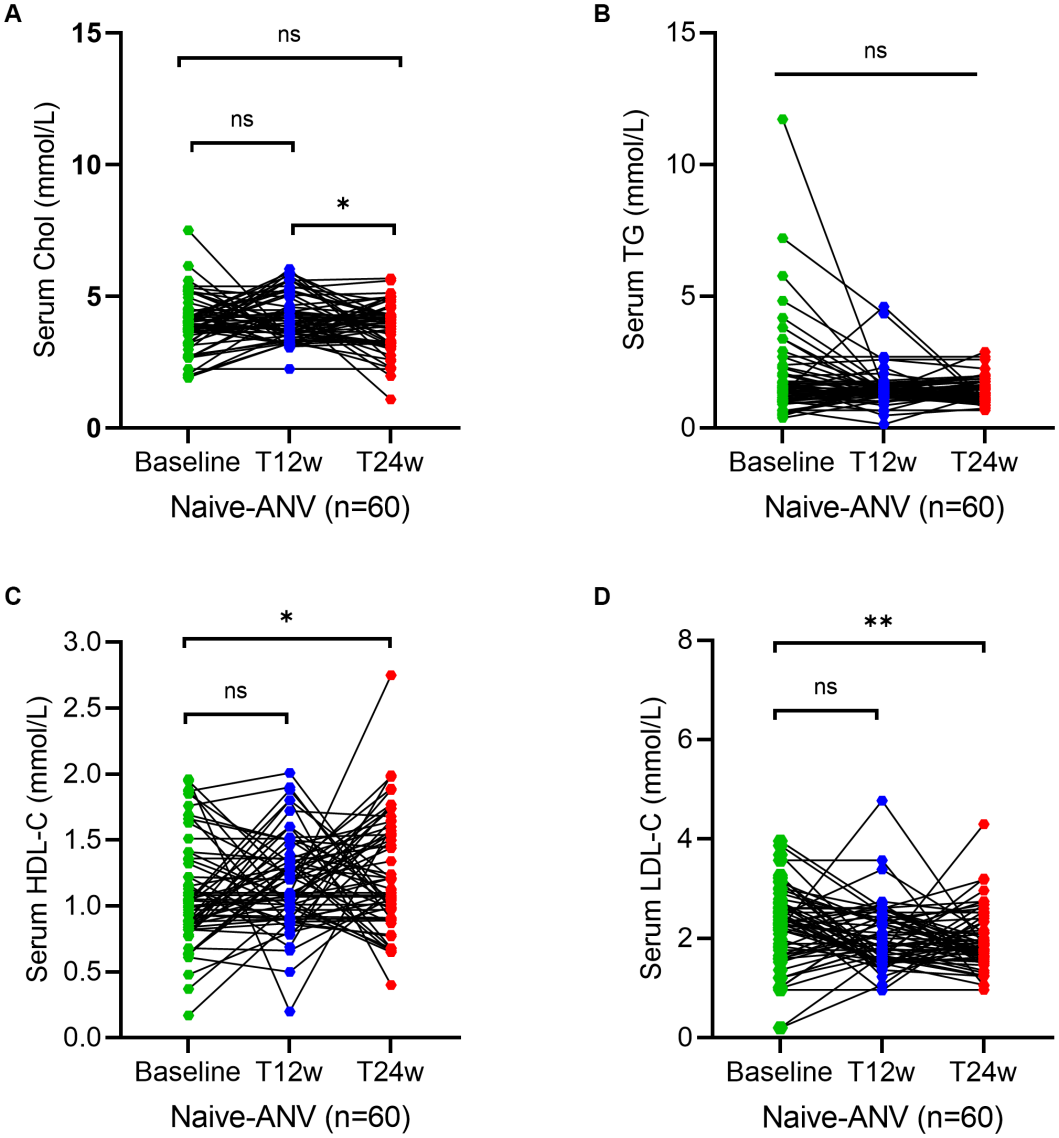
Longitudinal changes of lipid profile over time among treatment-naive patients receiving the ainuovirine (ANV)-based antiretroviral therapy. **(A–D)** Show dynamic changes in the serum levels of cholesterol (TC), triglycerides (TG), high-density lipoprotein cholesterol (HDL-C), and low-density lipoprotein cholesterol (LDL-C) at baseline, week 12 (T12w), and week 24 (T24w) following ANV-based treatment in naive patients with HIV infection, respectively. Each individual value and trend at baseline, T12w, and T24w are shown. A paired Wilcoxon signed-rank test was performed to determine the statistical significance in the serum lipid levels of patients comparing each of the two groups. Here, ns, *, **, ***, and **** indicate the *p*-values of >0.05, <0.05,<0.01, <0.001, and <0.0001.

**Figure 3:**
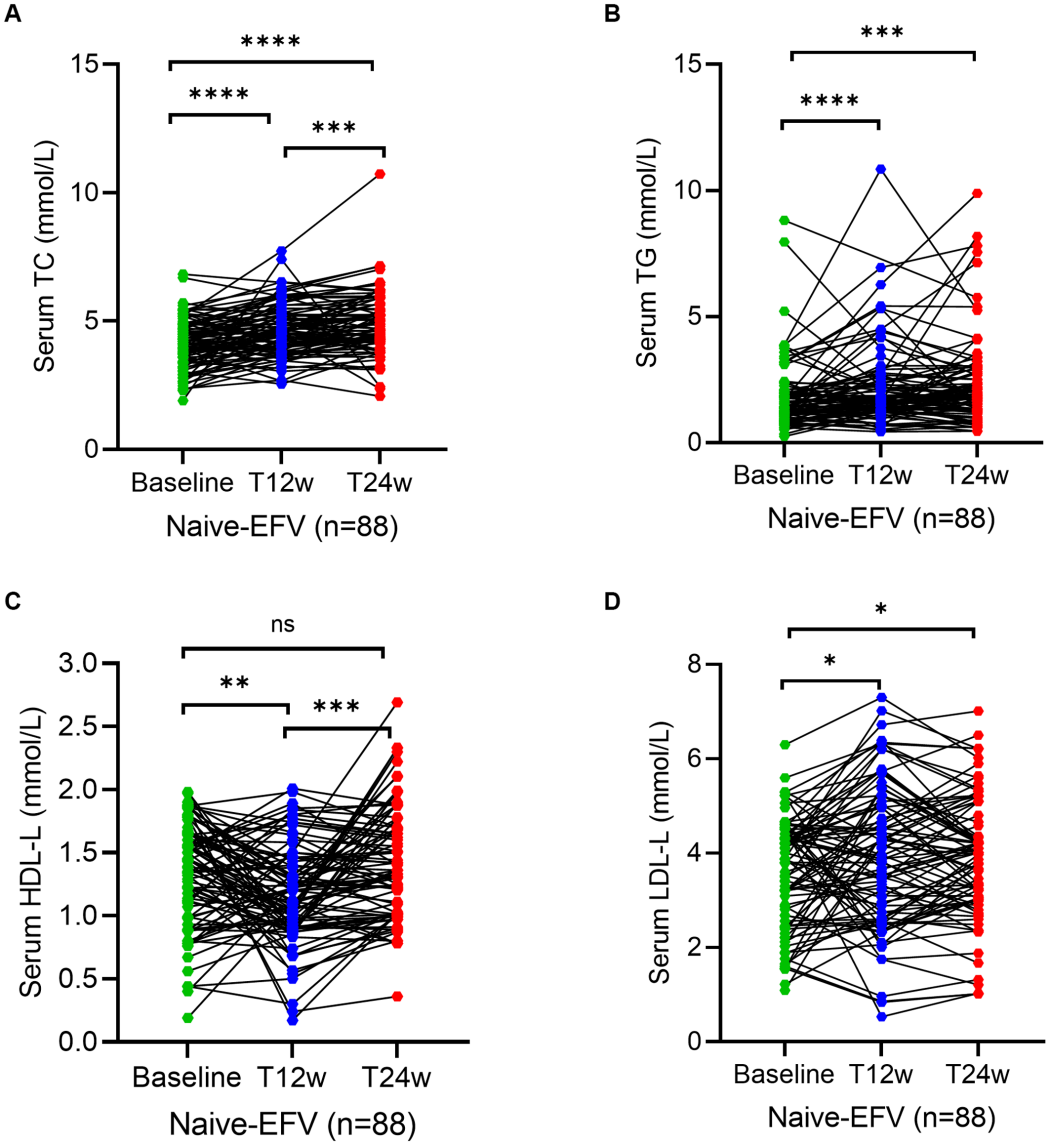
Longitudinal changes of lipid profile over time among treatment-naive patients receiving the efavirenz (EFV)-based antiretroviral therapy. **(A–D)** Show dynamic changes in the serum levels of cholesterol (TC), triglycerides (TG), high-density lipoprotein cholesterol (HDL-C), and low-density lipoprotein cholesterol (LDL-C) at baseline, week 12 (T12w), and week 24 (T24w) following EFV-based treatment in naive patients with HIV infection, respectively. Each individual value and trend at baseline, T12w, and T24w are shown. A paired Wilcoxon signed-rank test was performed to determine the statistical significance in serum lipid levels of patients comparing each of the two groups. Here, ns, *, **, ***, and **** indicate the p-values of >0.05, <0.05,<0.01, <0.001, and <0.0001, respectively.

To assess whether an ANV-based treatment better alters the status of serum lipid profiles in naive HIV/AIDS patients, we determined the levels of lipids using the assays as described in the Methods section. As shown, serum levels of TC and TG both remained relatively constant or even decreased throughout the 24 weeks of observation in naive HIV/AIDS patients treated with ANV-based regimens (Figures 2A,B). However, conversely, in the naive HIV/AIDS patients treated with EFV-based regimens, the serum levels of TC continuously increased at 12 weeks (4.5; IQR, 3.9–5.3 mmol/L) and 24 weeks following treatment (4.7; IQR, 4.2–5.7 mmol/L) from baseline (4.1; IQR, 3.4–4.8 mmol/L) (*p* < 0.0001) (Figure 3A), and the serum levels of TG kept increasing throughout not only 12 weeks (1.9; IQR, 1.3–2.6 mmol/L) (*p* < 0.0001) but also 24 weeks (1.9; IQR, 1.3–2.9 mmol/L) (*p* = 0.0001) after treatments from baseline level (median, 1.4; IQR, 1.1–2.0 mmol/L) (Figure 3B). In addition, in the naive-ANV group, the serum levels of HDL-C significantly elevated at 24 weeks (1.2; IQR, 1.0–1.6 mmol/L) (*p* = 0.02), but not at 12 weeks (1.1; IQR, 0.9–1.4 mmol/L) (*p* = 0.08) following treatment compared with the baseline (1.0; IQR, 0.8–1.3 mmol/L) (Figure 2C). Conversely, in the naive-EFV group, the serum levels of HDL-C significantly decreased at 12 weeks (1.1; IQR, 0.9–1.4 mmol/L) (*p* = 0.005) but not at 24 weeks (1.3; IQR, 1.0–1.7 mmol/L) (*p* = 0.39) following treatments from baseline (median, 1.4; IQR, 1.0–1.7 mmol/L) (Figure 3C); however, it still showed a downward trend. Again, in the naive-ANV group, the serum levels of LDL-C significantly decreased at 24 weeks (1.8; IQR, 1.6–2.3 mmol/L) (*p* = 0.02) but not at 12 weeks (1.9; IQR, 1.6–2.4 mmol/L) (*p* = 0.12) following treatment compared with the baseline (2.3; IQR, 1.7–2.7 mmol/L) (Figure 2D). However, the levels of LDL-C dramatically elevated at 12 weeks (3.9; IQR, 2.7–5.0 mmol/L) (*p* = 0.01) and 24 weeks (4.0; IQR, 3.1–4.8 mmol/L) (*p* = 0.03) following treatment compared with the baseline (3.8; IQR, 2.6–4.5 mmol/L) (Figure 3D). These results suggest that the degree of dyslipidemia that happened in the ANV-based groups is much lower than that in the EFV-based groups, indicating that ANV may be superior to EFV and, to some extent, may alleviate the progress of atherosclerosis in this population.

### 3.4 Lipid level changes after treatment in the treatment-experienced HIV/AIDS patients

**Figure 4:**
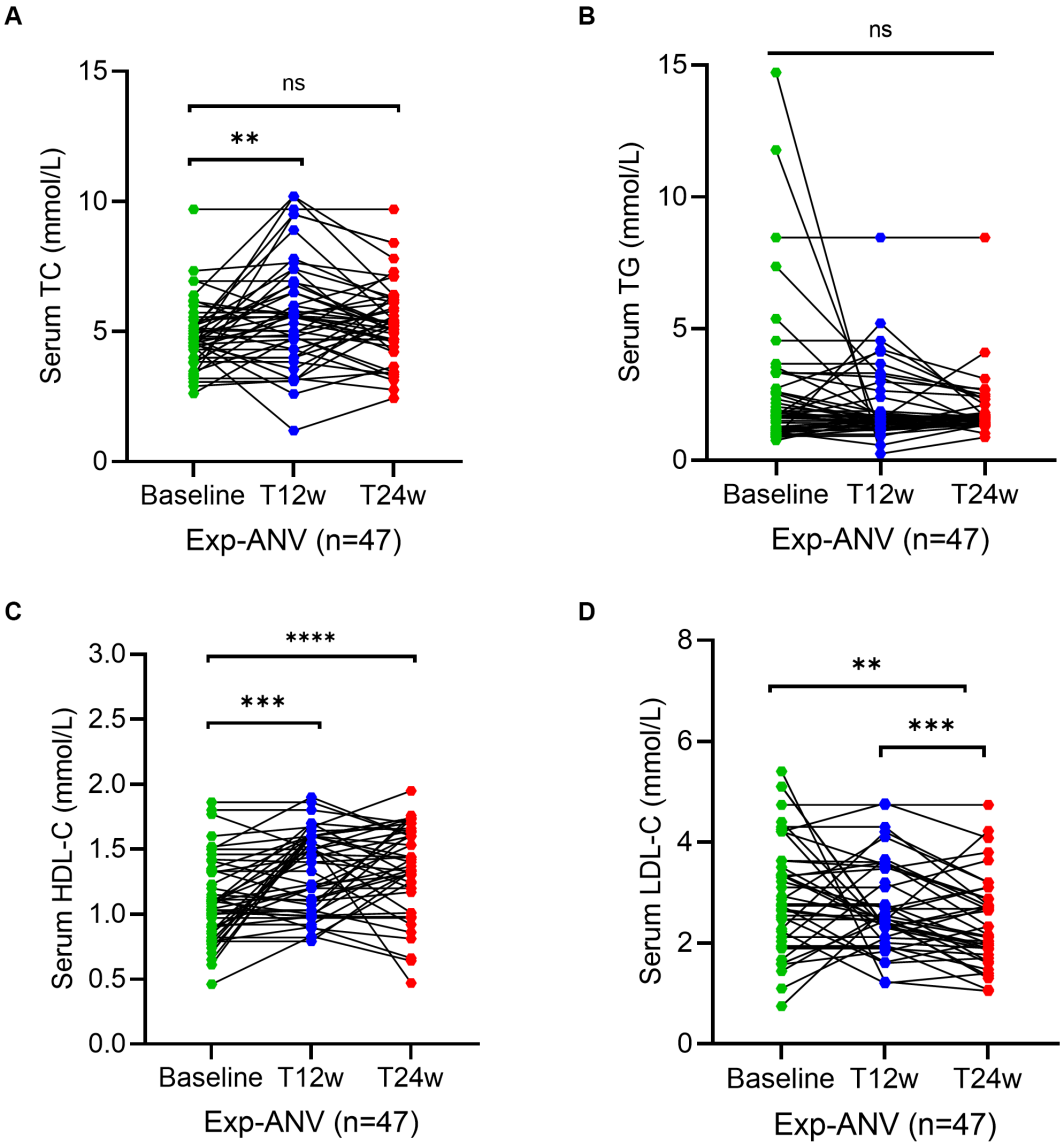
Longitudinal changes of lipid profile over time among treatment-experienced patients receiving the ainuovirine (ANV)-based antiretroviral therapy. **(A–D)** Show dynamic changes in the serum levels of cholesterol (TC), triglycerides (TG), high-density lipoprotein cholesterol (HDL-C), and low-density lipoprotein cholesterol (LDL-C) at baseline, week 12 (T12w), and week 24 (T24w) following EFV-based treatment in naive patients with HIV infection, respectively. Each individual value and trend at baseline, T12w, and T24w are shown. A paired Wilcoxon signed-rank test was performed to determine the statistical significance in the serum lipid levels of patients comparing each of the two groups. Here, ns, **, ***, and **** indicate the *p*-values of >0.05, <0.01, <0.001, and <0.0001, respectively.

**Figure 5:**
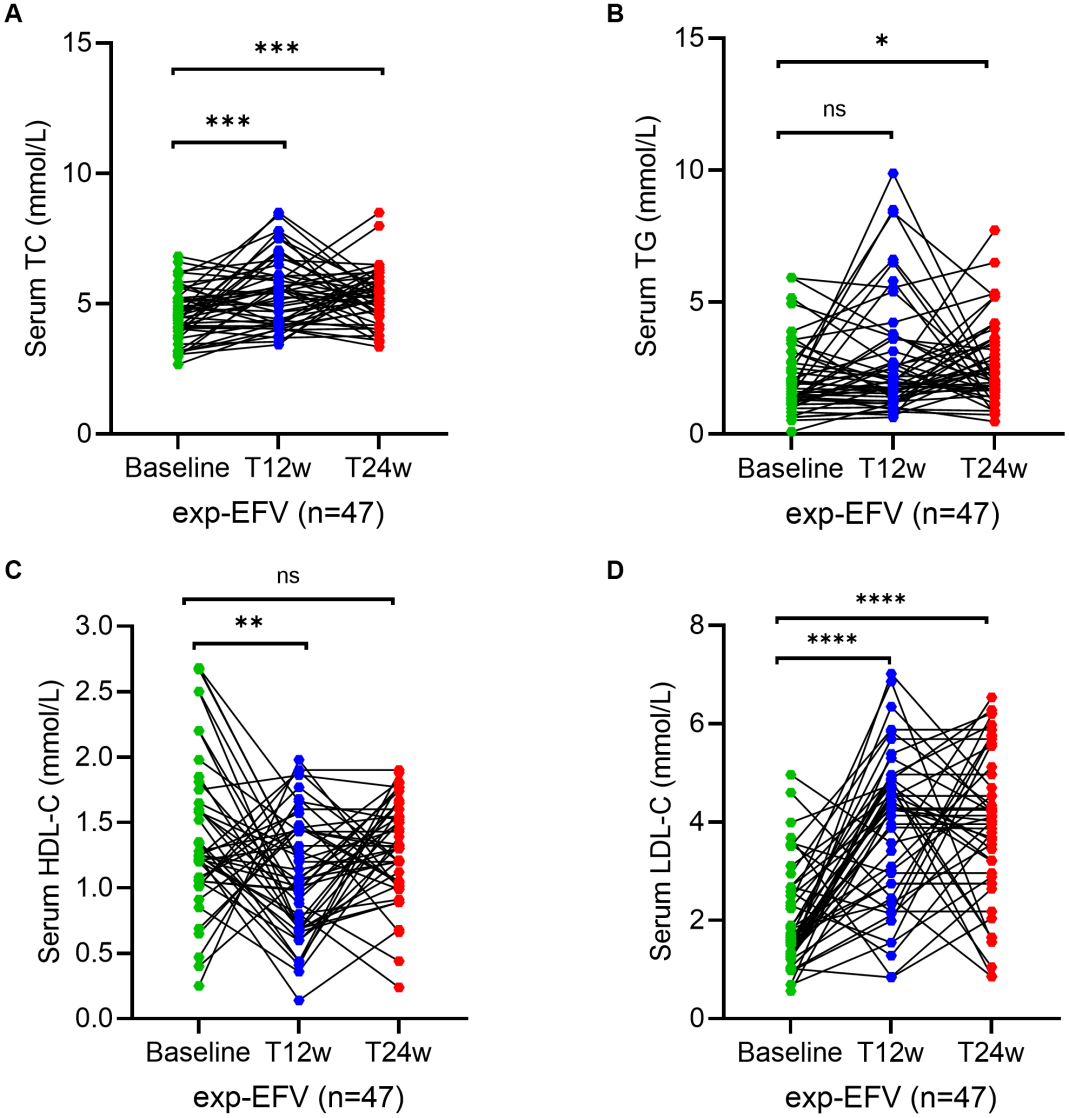
Longitudinal changes of lipid profile over time among treatment-experienced patients receiving the efavirenz (EFV)-based antiretroviral therapy. **(A–D)** Show dynamic changes in the serum levels of cholesterol (TC), triglycerides (TG), high-density lipoprotein cholesterol (LDL-C), and low-density lipoprotein cholesterol (LDL-C) at baseline, week 12 (T12w), and week 24 (T24w) following EFV-based treatment in naive patients with HIV infection, respectively. Each individual value and trend at baseline, T12w, and T24w are shown. A paired Wilcoxon signed-rank test was performed to determine the statistical significance in the serum lipid levels of patients comparing each of the two groups. Here, ns, *, and ** indicate the *p*-values of >0.05, <0.05, and <0.01, respectively.

To reconfirm the effectiveness of the ANV-based regimen, we determined the same parameters during the study period in treatment-experienced patients. Serum levels of TC at week 12 (5.6; IQR, 4.3–6.8 mmol/L) (*p* = 0.003) significantly increased in experienced HIV/AIDS patients treated with ANV compare with the baseline (4.7; IQR, 4.0–5.2 mmol/L), but not at week 24 (5.2; IQR, 4.6–6.2 mmol/L) (*p* = 0.06) (Figure 4A). However, in the exp-EFV group, the serum levels of TC persistently increased from the baseline (4.6; IQR, 4.0–5.2 mmol/L) to observational time week 12 (5.4; IQR, 4.2–6.1 mmol/L) (*p* = 0.0003) and week 24 (5.2; IQR, 4.6–5.9 mmol/L) (*p* = 0.0002) (Figure 5A). Conversely, the levels of TG remained constant or even slightly decreasing throughout the 24 weeks of observation in exp-ANV group (Figure 4B). However, in the exp-EFV group, the serum levels of TG dramatically increased over time following treatment of 24 weeks (2.1; IQR, 1.7–3.5 mmol/L) (*p* = 0.03), yet not at 12 weeks (2.0; IQR, 1.4–3.7 mmol/L) (*p* = 0.13) from the baseline (1.5; IQR, 1.2–2.7 mmol/L) (Figure 5B). Again, the serum levels of HDL-C in the exp-ANV group significantly elevated at both 12 weeks (1.4; IQR, 1.0–1.6 mmol/L) (*p* = 0.0002) and 24 weeks (1.4; IQR, 1.2–1.6 mmol/L) (*p* < 0.0001) following treatment compared with the baseline (1.1; IQR, 0.9–1.3 mmol/L) (Figure 4C), while the levels of HDL-C in the exp-EFV group went down at 12 weeks (1.1; IQR, 0.7–1.5 mmol/L) (*p* = 0.007) but not at 24 weeks (1.2; IQR, 1.0–1.5 mmol/L) (*p* = 0.08) following treatment (Figure 5C). Similarly, in the exp-ANV group, the serum levels of LDL-C continually and significantly decreased at 24 weeks (2.2; IQR, 1.9–2.9 mmol/L) (*p* = 0.009) but not at 12 weeks (2.5; IQR, 2.1–3.5 mmol/L) (*p* = 0.35) following treatment compared with the baseline (2.8; IQR, 2.1–3.6 mmol/L) (Figure 4D). However, levels of LDL-C dramatically increased at both 12 weeks (4.3; IQR, 2.5–4.9 mmol/L) (*p* < 0.0001) and 24 weeks (4.0; IQR, 2.9–5.1 mmol/L) (*p* < 0.0001) following treatment compared with the baseline (1.7; IQR, 1.3–2.7 mmol/L) in the exp-EFV group (Figure 5D).

### 3.5 Dynamic changes of dyslipidemia prevalence in the treatment-naive HIV/AIDS patients

**Figure 6:**
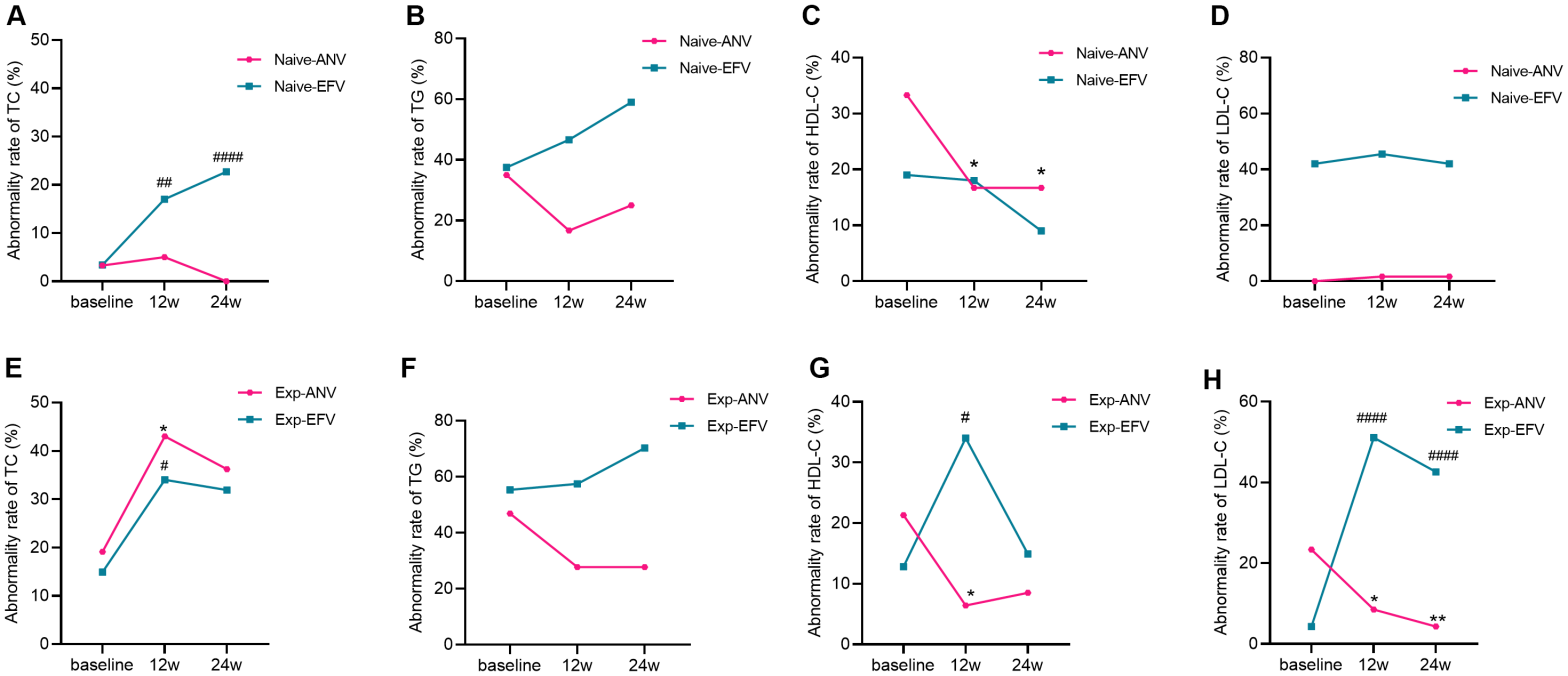
Dyslipidemia rate over time among treatment-naive and experienced patients receiving ainuovirine (ANV) or efavirenz (EFV)-based antiretroviral therapy. **(A–H)** Each individual value and trend at baseline, T12w, and T24w are shown. Fisher’s exact test or the chi-squared test was performed to determine the statistical significance in the dyslipidemia rate of each group compared with the baseline. Here, *and ^#^ indicate compared with baseline, *p* < 0.05; ** and ^##^ compared with baseline, *p* < 0.01; ^####^ compared with baseline, *p* < 0.0001.

Together, 50.0% of patients from the naive-ANV group and 55.6% from the naive-EFV group were classified as having at least one item of dyslipidemia at baseline based on the reference range from our assays. However, 41.6% of patients in the naive-ANV group and 73.9% in the naive-EFV group were classified as having one item of dyslipidemia after 24 weeks of treatment (*p* < 0.0001). After treatment, the proportion of patients with hypercholesterolemia (TC > 5.69 mmol/L) in the naive-EFV group increased from 3.4 to 22.7% (*p* < 0.0001) (Figure 6A), and those with hypertriglyceridemia (TG > 1.7 mmol/L) increased from 37.5 to 57.1% (*p* = 0.004) (Figure 6B). However, most of the patients with low HDL-C (< 0.9 mmol/mL) (Figure 6C) or high LDL-C (>4.14 mmol/L) (Figure 6D) did not change that much with significant differences (all *p* > 0.05). However, in the naive-ANV group, patients with low HDL-C decreased from 33.3 to 16.7% (*p* = 0.03). Conversely, the proportion of patients with hypercholesterolemia, hypertriglyceridemia, or high LDL-C did not increase but decreased somewhat, despite no apparently significant differences (*p* > 0.05).

### 3.6 Dynamic changes of dyslipidemia prevalence in the treatment-experienced HIV/AIDS patients

Interestingly, almost the same proportion of patients was classified as having one item of dyslipidemia at baseline in the experienced groups. Following 24 weeks of treatment, the prevalence of patients with dyslipidemia in the exp-ANV group decreased from 63.8 to 57.4% (*p* = 0.53); however, the proportion of patients with dyslipidemia in the exp-EFV group conversely rose from 59.6 to 85.1% (*p* = 0.006). In total, experienced patients in EFV group showed significantly raised cases of hypercholesterolemia (*p* = 0.03), and high LDL-C (*p* = 0.004) (Figures 6E,H) except for hypertriglyceridemia and low HDL-C (Figures 6F,G). Nevertheless, the exp-ANV group instead showed significantly reduced cases of hypertriglyceridemia, high LDL-C, and low HDL-C (all *p* < 0.05) (Figures 6F–H) except for hypercholesterolemia (Figure 6E). These results suggest that distinct ART regimens appear to promote different alterations in lipid metabolism.

## 4 Discussion

In this single-center study of antiretroviral-naive participants, it was found that the HIV-1 RNA <50 copies/mL rate of ANV-based treatment was inferior to that of EFV-based treatment at week 24, especially for participants with high viral loads (>100,000 copies/mL). The present study also demonstrated that both treatment-naive and treatment-experienced patients with HIV/AIDS undergoing ART exhibited a modest imbalance of their serum lipid profile. The ANV-based regimen, but not EFV-base regimen, even showed a trend of improvement in the serum levels of TG, HDL-C, and LDL-C. Therefore, our data confirmed initial observations suggesting a minimal effect of ANV combined with NRTIs on serum lipids in ART-naive and ART-experienced individuals infected with HIV. The study may address hard clinical endpoints, including virologic, immunologic, and metabolic evaluations.

Advances in the treatment of HIV/AIDS have led to significant improvements in both life expectancy and quality of life for the affected individuals. In this context, it is accompanied by gradually dominating long-term complications of HIV/AIDS patients, especially the appearance of chronic dyslipidemia, which is the most predominant and highly related risk of ASCVD. Consequently, it highlights the importance of the management of dyslipidemia. Specifically, lipid abnormalities are prevalent among PWH as a result of the infection itself, the effect of ART, and host factors as well. The varying prevalence of dyslipidemia among HIV-infected patients has been reported depending on the study and patient population, ranging from 20 to 80% (5).

Prior to the application of c-ART in 1996, studies demonstrated that HIV-infected patients exhibited decreased trends in TC and HDL-C, but showed elevated levels of plasma TG and free fatty acids in patients with AIDS (6). However, the use of protease inhibitor (PI), NRTIs, and NNRTIS have been associated with an increase in both TC and TG levels (7–14). Even though the underlying mechanism is not fully understood, it has been postulated as multifactorial, including a combination of elevations in circulating inflammatory mediators that induce both metabolic syndrome and immune reconstitution inflammatory syndrome, which can further modulate lipid metabolism (such as IFN-α and TNF), impaired TG clearance, and enhanced abnormal fat distribution in addition to the effects of individual ART (6, 15–18). Without doubt, other factors to consider with dyslipidemia are non-HIV factors, such as dietary choices, sedentary or low physical activity, concomitant medications, and other comorbidities, such as obesity, hypothyroidism, hypogonadism, and diabetes mellitus (19, 20). Isolated hypertriglyceridemia is rare in the setting of people with HIV on c-ART in the modern era, and the lipid profile usually shows mixed dyslipidemia. The effect of EFV on the dyslipidemia rate among patients is relatively weaker than lopinavir/ritonavir. In our study, EFV regimens were associated with increased TC and LDL-C levels, which is consistent with findings from previous studies (21–26). Moreover, both EFV and ANV regimens resulted in increased TC levels in treatment-experienced groups, but just slightly increased in treatment-naive groups. It may be attributed to a higher degree of hypercholesterolemia that existed in treatment-experienced individuals. However, in both treatment-naive and treatment-experienced patients, a dramatic elevation in the serum levels of TG was observed after employing EFV-based regimens, but not detected when using ANV-based regimens. Additionally, in our study, the levels of HDL-C, which are associated with decreased risk of ASCVD, were reduced moderately just in the naive-EFV group. In addition, beyond expectations, HDL-C levels were subversively increased in both naive and exp-ANV groups, resulting in more gradually reduced ratios of TC and TG to HDL-C in the ANV-based groups than those in the EFV-based controls (data not shown). Basically, the marked increase in these ratios is common in patients with advanced HIV disease and is indicative of the proinflammatory state, which is associated with metabolic disturbances such as insulin resistance and central adiposity (27). As such, an ANV-based regimen might reverse this situation, which may be explained by the ANV’s synergistic role with 3TC and TDF (28). Surprisingly, patients in the EFV-based groups were found to have a dramatic increase in LDL-C levels, which, however, were conversely and significantly decreased in the ANV-based groups, which is particularly in line with the general principle of “lower is better” for LDL-C (29). Overall, multiple favorable changes in the lipid profile, which may infer a low risk of cardiovascular disease, characterized the ANV arms.

In recent years, extensive efforts have been devoted to the development of alternative regimens and the elimination of the dyslipidemia effect during the c-CRT in PWH. DTG, an integrase inhibitor, is currently widespread as a first-line antiretroviral therapy globally, especially in developed countries. Moreover, improved lipid profiles were also detected in PWH with dyslipidemia receiving DTG-based regimens (30–32). However, weight gain and increased waist circumference were also observed and the price of DTG was >500% higher than EFV in many countries due to ongoing patent restrictions, which limited the widespread application of DTG in undeveloped countries (33–37). Meanwhile, in the large SINGLE study, levels of TG increased in both DTG and EFV groups (30, 32). Participants who switched to ANV in our study (exp-ANV group) clearly showed significant downward trends in their TG and LDL-C levels, but upward trends in their HDL-C levels through week 12 to week 24 following the treatment, which might be related to the enhanced lipid levels induced during the preceding treatment.

With respect to the use of a new drug, virological failure is the primary issue to be considered when clinicians propose changes in the treatment regimen for patients who are already under viral suppression. As indicated by the results of this study, in the treatment-naive individuals whose HIV-1 RNAs were >10,0000 copies/mL, the ANV-based regimen had a significantly higher rate of viral suppression than that of the EFV-based regimen, which can fill the gap that EFV is not recommended for patients with high viral load according to the WHO guidelines. Altogether, our data confirm that the use of ANV is safe and may exhibit an effective role in virologic suppression, immunologic response, and improvement of dyslipidemia.

There are still several limitations to this study. First, it was a single-arm, monocentric, and open-label study, with a relatively small sample size and a lack of female participants. Second, due to the relatively short follow-up duration, some effects may not have been detected yet. Third, this study did not carry out a thorough evaluation of lifestyle factors such as daily diet and exercise time, as well as the utility of lipid-lowering agents and previous treatments, which could have impacted the results of the study. Finally, it is important to note that the medication ANV, while approved for marketing, was exclusively prescribed to the intention-to-treat population. This approach may introduce selection bias, limiting the generalizability of the findings.

To conclude, we have shown a first real-world study that suggests that patients with HIV/AIDS receiving an ANV-based regimen have better control over their lipid profile than those who were provided with an EFV-based regimen, regardless of naive or experienced patients. ANV is well tolerated in real-life settings, and a longer-term follow-up study is still ongoing. These primary results suggest that ANV may provide an additional therapeutic option for PWH.

## Data availability statement

The original contributions presented in the study are included in the article/supplementary material, further inquiries can be directed to the corresponding author.

## Ethics statement

The studies involving humans were approved by Institutional Review Boards of Chinese Center for disease control and prevention. The studies were conducted in accordance with the local legislation and institutional requirements. The participants provided their written informed consent to participate in this study.

## Author contributions

QZ: Conceptualization, Methodology, Software, Writing – original draft. ZC: Conceptualization, Data curation, Investigation, Methodology, Writing – original draft. YW: Data curation, Writing – review & editing. ST: Data curation, Investigation, Writing – review & editing. YP: Data curation, Writing – review & editing. YL: Investigation, Writing – review & editing. GC: Data curation, Investigation, Writing – review & editing. AB: Writing – review & editing. SW: Funding acquisition, Supervision, Writing – review & editing. MW: Investigation, Supervision, Funding acquisition, Writing – review & editing.
